# Linked region detection using high-density SNP genotype data via the minimum recombinant model of pedigree haplotype inference

**DOI:** 10.1186/1471-2105-10-216

**Published:** 2009-07-15

**Authors:** Lusheng Wang, Zhanyong Wang, Wanling Yang

**Affiliations:** 1 Department of Computer Science, City University of Hong Kong, Hong Kong, PR China; 2 Department of Paediatrics & Adolescent Medicine, LKS Faculty of Medicine, The University of Hong Kong, 21 Sassoon Road, Hong Kong, PR China

## Abstract

**Background:**

With the rapid development of high-throughput genotyping technologies, efficient methods for identifying linked regions using high-density SNP genotype data have become more and more important. Recently, a deterministic method that works very well on SNP genotyping data has been developed (Lin et al. Bioinformatics 2008, 24(1): 86–93). However, that program can only work on a limited number of family structures. In particular, the results (if any) will be poor when the genotype data for the whole chromosome of one of the parents in a nuclear family is missing.

**Results:**

We have developed a software package (LIden) for identifying linked regions using high-density SNP genotype data. We focus on handling the case where the genotype data for the whole chromosome of one of the parents in a nuclear family is missing. We use the minimum recombinant model for haplotype inference in pedigrees. Several local optimization algorithms are used to infer the haplotype of each individual and determine the linked regions based on the inferred haplotype data. We have developed a more flexible method to combine nuclear families to further refine (reduce the length of) the linked regions.

**Conclusion:**

Our new package (LIden) is efficient software for linked region detection using high-density SNP genotype data. LIden can handle some important cases where the existing programs do not work well. In particular, the new package can handle many cases where the genotype data of one of the two parents is missing for the entire chromosome. The running time of the program is *O*(*mn*), where *m *is the number of members in the family and *n *is the number of SNP sites in the chromosome. LIden is specifically suitable for handling big sized families. This research also demonstrates another practical use of the minimum recombinant model for haplotype inference in pedigrees.

The software package can be downloaded at .

## Background

With the completion of the human genome sequencing project and the development of HapMap project [1], our understanding of human genomic sequences has been extended dramatically. Due to the development of SNP genotyping technology, genotyping of hundreds of thousands of single nucleotide polymorphism (SNP) markers in a high-throughput format has become a routine job in many labs.

Compared to classical genotyping methods mainly using microsatellite markers, SNP genotyping is faster and easier. It provides complete coverage of the genome and much more information on covered regions. Linkage analysis is a method to identify genomic regions that cosegregate with an inherited disease in a family and to facilitate the eventual identification of the mutation in that region causing the disease. Leykin et al. in [2] and Sellick et al. in [3] demonstrated that high-density SNP genotype data, such as that from microarrays, can be used for large-scale and cost-effective linkage analysis. The main reason is that there will be a sufficient number of informative markers between any two recombination points and thus the allele sharing status among the family members can be precisely determined. Therefore, efficient programs are highly demanded for allele sharing determination that work on a large number of markers and big sized families.

Classical linkage analysis methods are designed for sparse microsatellite markers. They are mainly based on two algorithms, the Elston-Steward algorithm that is limited by the number of total markers used [4] and the Lander-Green algorithm that is limited by the total number of individuals in a family [5]. As a result, they either cannot handle genotype data based on large number of SNPs at all or they cannot handle families of a large size, especially together with large numbers of genotyped SNPs, due to memory constraint.

Recently, a deterministic method that works very well on SNP genotyping data [6] has been developed. This was one of a series of efforts to develop software that is particularly suitable for SNP genotyping data and runs in time linear to both the number of SNP sites and the number of family members. However, the program in [6] can only work on a limited number of family structures. Here we use the minimum recombinant model for haplotype inference in pedigrees and develop a set of algorithms to minimize the total number of recombinants and produce a software package that works on a much wider range of family structures. Extensive simulations on Affymetrix Human Mapping 50 K/250 K GeneChips showed that the new package can correctly identify the linked regions on a wide range of family structures. In particular, the new package outperforms the old program in many important cases where the genotype data of one of the parents is missing on the entire chromosome. This research also demonstrates another practical use of the minimum recombinant model for haplotype inference in pedigrees.

## Implementation

We use the minimum recombinant model to infer the haplotype configuration for all the family members. In 2002, Qian and Beckman [7] proposed a model to reconstruct haplotype configurations from genotype data in a pedigree under the Mendelian law of inheritance. In this model, the resulting haplotype configurations should have the minimum number of recombinants (i.e. recombination events).

### Minimum Recombinant Haplotype Configuration

Given a pedigree and the genotype information for each member, the object is to find a haplotype configuration such that the total number of recombinants in the whole pedigree is minimized.

The problem is called Minimum Recombinant Haplotype Configuration (MRHC). The MRHC problem was proved to be NP-hard by Li and Jiang [8]. Lots of algorithms have been proposed. Some algorithms run in time exponential in terms of the number of SNP sites and some algorithms run in time exponential in terms of the number of family members [9]. An integer linear programming approach was proposed to handle incomplete genotype data [10].

Linkage analysis aims to identify regions whose allele is shared by all or most diseased members in a family but by none or few normal family members. In dominant inheritance situations, sharing of one mutation allele can cause a disease phenotype. In recessive cases, sharing of two disease alleles in that region is necessary for there to be a diseased status. We will first design algorithms to infer the allele sharing status with minimum recombinants and then use an algorithm to find the linked regions(regions shared by all or most of the diseased individuals but not shared by any normal individuals) by possibly changing the inferred allele sharing status.

## Algorithm

Our package contains a set of (heuristic) algorithms to handle various kinds of situations and sometimes for one case, we use two (local optimization) algorithms to iteratively refine the output. Before we discuss the algorithms, we give the basic data structures used in the package.

### The basic data structures

For each individual in the family, we use five arrays: **genotype**, **paternal-h**, **maternal-h**, **which-p **and **which-m **to store the information. The possible values for each element in the arrays are given in Table 1. The genotype value of individual *I *at site *i *is in **I. genotype [i]**, which is {*A, A*}, {*A, B*} or {*B, B*}. The haplotype value of individual *I *from the father at site *i *is in **I.paternal-h [i]**, which is either *A *or *B*. Similarly, the haplotype value of individual *I *at site *i *from the mother is in **I. maternal-h [i]**, which is either *A *or *B*. For each individual, there are two (paternal and maternal) copies of haplotypes. We use 0 and 1 to distinguish them. **I.which-p [i] **can be 0 or 1.

**Table 1:** The possible values of each element in the five arrays.

Array Name	Possible Values of each element
genotype	{*AA*}, {*AB*}, {*BB*}

paternal-h	*A*, *B*

maternal-h	*A*, *B*

which-p	0, 1

which-m	0, 1

**I.which-p [i] = 0 **indicates that the haplotype **I.paternal-h [i] **of individual *I *is from his/her father's 0-th haplotype and **I.which-p [i] = 1 **indicates that the haplotype **I.paternal-h [i] **of individual *I *is from his/her father's 1-th haplotype. Similarly, **I.which-m [i] = 0 **indicates that the haplotype **I. maternal-h [i] **of individual *I *is from his/her mother's 0-th haplotype and **I.which-m [i] = 1 **indicates that the haplotype **I. maternal-h [i] **of individual *I *is from his/her mother's 1-th haplotype. The main purpose here is to keep a record of which grandparent the allele came from.

### The algorithms for nuclear families with data available for both parents

**Figure 1:**
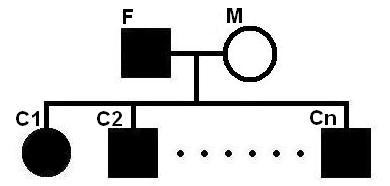
**A family with *n *children**. A nuclear family with two parents and n children *C*_1_, *C*_2_,..., *C*_*n*_. A box represents a male individual and a circle represents a female individual. The filled circles/boxes indicate diseased individuals and the open circles/boxes indicate normal individuals. This setting applies to all the figures in this paper.

Let us consider a nuclear family with two parents and *n *children *C*_1_, *C*_2_,..., *C*_*n*_. The pedigree is shown in Figure 1. A box represents a male individual and a circle represents a female individual. The filled circles/boxes indicate diseased individuals and the open circles/boxes indicate normal individuals. This setting applies to all the figures in this paper. To handle nuclear families with both parents, we use two algorithms, the *basic algorithm *and the *horizontal local optimization algorithm*.

### The basic algorithm

In our basic algorithm, we consider a site at a time. Suppose that *paternal *[*i*], *maternal *[*i*], *which*-*p *[*i*] and *which*-*m *[*i*] of each individual at site *i *have been fixed. For site *i *+ 1, there are (at most) 4 different haplotype configurations of the two parents fitting the given genotype data. For each of the 4 haplotype configurations of the two parents, we can fix *C*_*j*_*.paternal-h *[*i *+ 1], *C*_*j*_*.maternal-h *[*i *+ 1], *C*_*j*_*.which-p *[*i *+ 1] and *C*_*j*_*.which-m *[*i *+ 1] for every child *C*_*j *_(*j *= 1, 2,..., *n*) such that the number of break points of child *C*_*j *_between site *i *and *i *+ 1 is minimized. Note that, for each child *C*_*j*_, the number of breakpoints between site *i *and site *i *+ 1 could be 0, 1 or 2. When two choices are equally good for child *C*_*k*_, we arbitrarily select one. We then choose one of the 4 haplotype configurations of the parents at site *i *+ 1 such that the total number of break points for *all *the *n *children between site *i *and site *i *+ 1 is minimized. It is worth pointing out that the basic algorithm here considers *all *the children site by site while the old algorithm in [6] considers all the sites for every child and then handles the children of the nuclear family one by one. Clearly, the quality of the solution at site *i *+ 1 heavily depends on the quality of the solution at site *i*. Thus, we will first use a method to select a starting site that generates a good solution and then we use the algorithm to compute the solution to the left and right ends of the chromosome, respectively.

#### Finding a good starting site

Here we try to find a site, where the haplotypes for all individuals are uniquely determined according to the given genotype data. This can be done if both genotypes of the parents are "AB", and every child's genotype is "AA" or "BB". If there is no such site, we look for the "second best" site, where some of the individuals' haplotypes can be partially fixed. The second best site is a site where the genotype of one parent is "AB", and the genotype of each child is "AA" or "BB". In this case, we can uniquely determine the haplotypes for all children, but partially determine the inheritance information, i.e., one parent has genotype "AA" ("BB") and the inherited "A" ("B") of a child from this parent could be any one of "AA" ("BB"). For this case, we arbitrarily give a solution which fits the genotype data. The third best site is a site where both genotypes of father and mother are "AB", and the genotypes of some (but not all) children are "AA" or "BB". In this case, we can fix the haplotypes of those children with "AA" or "BB" correctly. But for a child with genotype "AB", we have a risk of making mistakes. Again, in this case, we arbitrarily give a solution which fits the genotype data. The worst case is that both genotypes of father and mother are "AA" or "BB". In this case, we cannot fix the inheritance source for any child. In practice, we can always find a site which is one of the first three types.

Note that there is no guarantee that we can always find a starting site with uniquely determined haplotypes. When the solution on the starting site is wrong, our algorithm may produce a short segment with many breakpoints. Whenever such a segment is found, our algorithm will re-calculate the solution of the segment using the reverse order and thus another starting point.

#### The horizontal local optimization algorithm

After we obtain a haplotype solution from the basic algorithm, we can look at three individuals, two parents and one of their children *C*_*j*_, at a time. Assuming that the haplotypes of the two parents are fixed, the number of break points in *C*_*j *_might be further reduced if we change the haplotypes of *C*_*j*_. This is due to the existence of multiple solutions at a site and the fact that the haplotype solution at site *i *heavily depends on that of its previous site. Thus, we use an algorithm that can give a haplotype of *C*_*j *_with minimum number of break points when the haplotypes of the two parents are fixed (by the basic algorithm). In this way, the total number of break points can be reduced. Let *D*^*pq*^(*i*) be the minimum number of breakpoints of *C*_*j *_for the first *i *sites such that at site *i *the paternal haplotype is from the *p*-th haplotype of the father and the maternal haplotype is from the *q*-th haplotype of the mother, where *p *= 0, 1 and *q *= 0, 1. Then *D*^*pq*^(*i *+ 1) can be computed based on *D*^00 ^(*i*), *D*^01 ^(*i*), *D*^10 ^(*i*) and *D*^11 ^(*i*). For example, the value of *D*^00 ^(*i *+ 1) can be one of *D*^00 ^(*i*), *D*^01 ^(*i*) + 1, *D*^10 ^(*i*) + 1 or *D*^11 ^(*i*) + 2. We can check each of the cases and see if the genotype of *C*_*j *_at site *i *+ 1 can fit each of the four configurations under the Mendelian law of inheritance. Among all the possible configurations, we choose the one corresponding to the minimum value. The running time of the algorithm is *O*(*n*), where *n *is the number of sites in the chromosome.

We apply the horizontal local optimization algorithm to each of the *n *children in the nuclear family one by one in an arbitrarily fixed order. (The order among the children does not affect the results.)

#### The whole algorithm for a nuclear family

Consider a nuclear family containing two generations. For a segment from position *i *to position *i *+ *k*, we can use the basic algorithm in two ways, i.e., from left to right or from right to left. We may get different solutions since the starting points are different. After we obtain a solution using the basic algorithm, we can use the horizontal local optimization algorithm to further improve the solution. The whole algorithm is as follows:

1. Find a good starting point as described.

2. Use the basic algorithm to get a solution for all individuals in the nuclear family.

3. Identify a short segment with a large number of breakpoints and apply the basic algorithm to this segment to re-calculate using the inverse order (thus a different starting point). If we can reduce the total number of break points then we use the new solution for this segment.

4. Use the horizontal local optimization algorithm for each child to refine the solution.

### The algorithms for nuclear families with data available for single parents

Now, we consider the case, where the genotype data of one of the parents in the nuclear family is unknown over the entire chromosome. To handle this case, the basic idea is similar to that for nuclear families with data available for both parents. For the basic algorithm, we guess the haplotype of the unknown parent whenever needed. Since each individual has two copies of haplotypes on each chromosome, there are four different haplotype configurations at each site. The two haplotypes for an individual can be AA, AB, BA, and BB. Thus, we can modify the basic algorithm to handle this case, where the genotype data for one parent is missing. In the basic algorithm, instead of considering at most 4 configurations of the two parents, we consider at most 8 configurations, where the unknown parent has 4 configurations, and the known parent has at most 2 configurations. Similarly, for each of the 8 configurations of the two parents, we can fix *C*_*j*_*.paternal*-*h *[*i *+ 1], *C*_*j*_*.maternal*-*h *[*i *+ 1], *C*_*j*_*.which*-*p *[*i *+ 1] and *C*_*j*_.*which*-*m *[*i *+ 1] for every child *C*_*j *_(*j *= 1, 2,..., *n*) such that the number of breakpoints of child *C*_*j *_between site *i *and *i *+ 1 is minimized. The rest of the algorithm remains the same.

### The algorithm for a family with three or more generations

To handle a family with three generations, we will view the set of all individuals in the first and second generations as a nuclear family which is referred to as the *main *nuclear family. For any child *C*_*j *_in the main nuclear family, if *C*_*j *_has his/her own children (third generation individuals), then we will view *C*_*j *_as a *super *child representing the *second generation nuclear family *including *C*_*j*_, *C*_*j*_'s spouse and all their children. The basic algorithm for a family with three generations is similar to the basic algorithm for a nuclear family. Here we focus on the main nuclear family and give some special treatment for super children.

### The basic algorithm for a family with three generations

Again, we consider a site at a time. Suppose that *paternal*-*h *[*i*], *maternal*-*h *[*i*], *which*-*p *[*i*] and *which*-*m *[*i*] of each individual in the main nuclear family for site *i *are fixed. Let us consider site *i *+ 1. If the genotype data is known for both parents, there are (at most) 4 different haplotype configurations of the two parents (first generation individuals) fitting the given genotype data. If the genotype data of a parent is missing, there are at most 8 different haplotype configurations of the two parents. Let *C*_1_, *C*_2_,..., *C*_*n *_be the *n *children in the second generation and some of them might be super children. For each possible haplotype configurations of the two parents (first generation individuals), we try to fix *C*_*j*_*.paternal*-*h *[*i *+ 1], *C*_*j*_*.maternal*-*h *[*i *+ 1], *C*_*j*_*.which*-*p *[*i *+ 1] and *C*_*j*_*.which*-*m *[*i *+ 1] for every child *C*_*j *_(*j *= 1, 2,..., *n*) as follows:

A1: if *C*_*j *_is not a super child, we fix *C*_*j*_*.paternal*-*h *[*i *+ 1], *C*_*j*_*.maternal*-*h *[*i *+ 1], *C*_*j*_*.which*-*p *[*i *+ 1] and *C*_*j*_*.which*-*m *[*i *+ 1] such that the number of breakpoints of child *C*_*j *_between site *i *and *i *+ 1 is minimized. Note that for each child *C*_*j*_, the number of breakpoints between site *i *and site *i *+ 1 could be 0, 1 or 2.

A2: if *C*_*j *_is a super child, we fix *C*_*j*_*.paternal*-*h *[*i *+ 1], *C*_*j*_*.maternal*-*h *[*i *+ 1], *C*_*j*_*.which*-*p *[*i *+ 1] and *C*_*j*_.*which*-*m *[*i *+ 1] such that the number of breakpoints  in the second generation nuclear family *C*_*j *_represented by *C*_*j *_between site *i *and *i *+ 1 is minimized. Here if the genotype data for both *C*_*j *_and *C*_*j*_'s spouse is given, there are at most two choices for each of *C*_*j *_and *C*_*j*_'s spouse. We can call the basic algorithm to get . Note that  could be greater than 2.

Again, when several choices are equally good for child *C*_*k*_, we arbitrarily select a choice.

Among all the possible haplotype configurations of the parents (first generation individuals) at site *i *+ 1, we select one such that the total number of breakpoints for *all *the individuals in the three-generation family between site *i *and site *i *+ 1 is minimized. This process can be used recursively to handle more than three generations. In fact, for our package, there is no limit on the number of generations in the family. Clearly, if the genotype data of both parents in all nuclear families is known, the running time of the basic algorithm is *O*(*mn*), where *m *is the total number of individuals in the whole family and *n *is the number of SNP sites in the chromosome.

### Dealing with missing individuals in the second generation

In this subsection, we deal with the cases where the genotype data for one of the parents in a second generation nuclear family is missing. The algorithm is similar to the basic algorithm for a family with three generations. Let *C*_*j *_be a super child. Two cases arise.

#### The genotype data for *C*_*j*_'s spouse is missing

For this case, when we try to fix *C*_*j*_*.paternal*-*h *[*i *+ 1], *C*_*j*_.*maternal*-*h *[*i *+ 1], *C*_*j*_*.which*-*p *[*i *+ 1] and *C*_*j*_*.which*-*m *[*i *+ 1] such that the number of breakpoints in the second generation nuclear family represented by *C*_*j *_between site *i *and *i *+ 1 is minimized as in A2, we have to guess the haplotyes of *C*_*j*_'s spouse by trying all possible haplotypes *AA*, *AB*, *BA *and *BB*. When the genotype data for *C*_*j*_'s spouse is given, there are two choices. This will slightly slow down the program. Moreover, if there are more than one second generation nuclear families missing the super child's spouse's genotype data, the speed will not be affected too much, since children in the second generation are processed one by one.

#### The genotype data for *C*_*j *_is missing

For this case, when we try to fix *C*_*j*_*.paternal *[*i *+ 1], *C*_*j*_*.maternal *[*i *+ 1], *C*_*j*_*.which*-*p *[*i *+ 1] and *C*_*j*_*.which*-*m *[*i *+ 1] such that the number of breakpoints in the second generation nuclear family represented by *C*_*j *_between site *i *and *i *+ 1 is minimized as in A2, we have to guess the haplotyes of *C*_*j *_by trying all possible haplotypes *AA*, *AB*, *BA *and *BB *without genotype data. Again, the running time is still *O*(*m *× *n*) since children in the second generation are processed one by one.

#### Remarks

For the algorithm in [6], a top-down approach is used to deal with three-generation families. The algorithm processes nuclear families (with two generations) one by one from the top to the bottom. The old approach cannot give good solutions when the size of the main nuclear family is small. The reason is that the quality of solutions heavily depends on the sizes of nuclear families and if the size of the main nuclear family is small, the obtained haplotypes for the (super) children in the second generation could be wrong and this wrong information will be passed to the processing of the third generation. A better strategy is to work on big nuclear families first. However, even this strategy is not as good as our approach here since we do not fix the solution of super children in the second generation. We have observed that the inferred haplotypes for big sized second generation families such as two parents and 5 children could be wrong though the breakpoint positions are very accurate. If the wrong haplotyes are used to handle other nuclear families, the whole linked region could be missed.

The current version of our program works for any number of generations. It can handle the case, where the genotype data for one of a couple is missing.

### Genotype data error correction

For large-scale SNP genotyping, a certain number of experimental errors is unavoidable. We observe that when genotype data errors occur, the inferred haplotypes contain many breakpoints that are close to each other. In order to get the correct allele sharing status, our algorithm will simply delete both breakpoints that are within *x *SNP sites. We suggest setting the value of *x *based on the error rate. When the error rate is smaller than 0.1%, *x *= 5. When the error rate is between 0.1% and 0.3%, *x *= 8. When the error rate is between 0.3% and 0.5%, *x *is set to be 10. When the error rate cannot be estimated, we simply use *x *= 8.

### Identifying mutation regions

After obtaining the inferred haplotype data for all the individuals in the family, we can find possible mutation regions by looking at the SNP sites one by one. The possible mutation regions should be regions shared by all or most of the diseased family members (considering phenocopy) but none or few of the normal family members (considering penetrance). Those regions are reported as suspected mutation regions. Due to the existence of multiple solutions and haplotype inference error, it is possible that the reported regions do not completely overlap the true mutation region. In our algorithm, we use a subroutine to extend the suspected mutation regions site by site in both directions, where we can revise the haplotype allele such that the allele is shared by diseased family members, but not shared by the normal family members. Unlike the program in [6], here we have to extend in both directions.

The current version of the package reports all the possible regions. The users are asked to input the maximum number of normal individuals to be allowed to share the mutation allele (allowing for penetrance) and the maximum number of diseased individuals in the family to be allowed not to share the potential mutation region (allowing for phenocopy).

## Results and Discussion

In this section, we will test our software package using simulated data. We have considered a wide range of pedigree structures.

### Generating haplotype data using the Chi-square model

In order to test the program, we generated haplotype datasets based on the Chi-square model to see if our program can infer the haplotype data correctly from the corresponding genotype data. The founder haplotypes were obtained from real datasets (Affymetrix Human Mapping 50 K/250 K GeneChips [11]), and children haplotypes were generated through random inheritance of paternal/maternal alleles using the Chi-square model for recombination with m equals 4 [6,12,13] and according to male/female averaged genetic map for chromosome 1 downloaded from HapMap [14]. The simulation process is identical to that of [6,15]. When disease status was considered, a mutation was randomly assigned to be close to a SNP site (called *mutation site*), and the diseased individuals were forced to inherit the mutation strand and the normal individuals were forced not to inherit the mutation strand. This process is done generation by generation.

### Nuclear families with data available for both parents

Let us consider a nuclear family as shown in Figure 1. When the genotype data for both parents is given, the results are shown in Table 2. Here we test the cases, where there are 2, 3,..., 6 children in the nuclear family. The results are listed in column 2, 3,..., 6, respectively. We have selected individuals to form 95 couples as in [6]. We have done experiments on 285 datasets (three times for each couple) and calculated the average. For all the simulations, the number of data sets is a multiple of 95. Each cell in the table contains two values. The first one is by our program and the one in brackets is by the program in [6]. A breakpoint is correctly inferred if the inferred breakpoint is within 20 SNPs away from the real location. From Table 2, we can see that all the elements in the row "overlap/real" are 1 indicating that our package can always identify the whole mutation region. The elements in the row "overlap/found" are about 0.97 or 0.96 indicating that the sizes of the reported regions are slightly larger than (almost identical to) that of the real mutation regions. The values of recall and precision are also very good when there are more than two children. The values in brackets are by the program in [6]. We can see that both programs perform very well in those cases.

**Table 2:** The experimental results for nuclear families when the genotype data for both parents is available.

	2 children	3 children	4 children	5 children	6 children
No. of Breakpoints	11.11 (10.59)	15.63 (15.50)	21.32 (21.28)	26.61 (26.60)	31.66 (31.60)

precision	0.47 (0.50)	0.93 (0.98)	0.95 (0.97)	0.98 (0.99)	0.98 (0.99)

recall	0.47 (0.48)	0.91 (0.96)	0.94 (0.97)	0.96 (0.98)	0.96 (0.98)

length of real linked region (cM)	76.66	59.51	40.82	35.97	29.90

length of found linked region (cM)	78.70 (79.51)	60.79 (60.77)	42.15 (42.13)	37.00 (37.01)	30.97 (30.99)

overlap/real	1.00 (1.00)	1.00 (1.00)	1.00 (1.00)	1.00 (1.00)	1.00 (1.00)

overlap/found	0.97 (0.96)	0.97 (0.97)	0.96 (0.96)	0.96 (0.96)	0.95 (0.95)

linked region recovery	1.00 (1.00)	1.00 (1.00)	1.00 (1.00)	1.00 (1.00)	1.00 (1.00)

The row "No. of Breakpoints" indicates the number of breakpoints in the whole family. The row "precision" is the ratio of the number of correctly inferred breakpoints to the total number of inferred breakpoints. The row "recall" is the ratio of the number of correctly inferred breakpoints to the number of real breakpoints. The row "length of real linked region (cM)" is the length of the linked region generated in the simulation. The row "length of found linked region (cM)" is the length of the linked region calculated by our program. The row "overlap/real" is the length of the region shared by both the real linked region and the inferred linked region divided by the length of the real linked region. The row "overlap/found" is the length of the region shared by both the real linked region and the inferred linked region divided by the length of the inferred linked region. The row "linked region recovery" is the number of times that the inferred linked region contains the mutation site divided by the total number of experiments. In each cell, the first value is by our program and the one in brackets is by the program in [6].

### Nuclear families with data available for single parents

Let us consider nuclear families with a single parent. In this case, the genotype data for the other parent is missing. About half of the children are diseased. There are two cases: (1) the available parent is diseased and (2) the available parent is normal. The results are shown in Table 3 and Table 4, respectively. Each cell in the tables contains two values. The first one is by our program and the one in brackets is by the program in [6]. We consider the cases where there are 2, 3,..., 6 children. For both cases, we can see that for our program, the precision and the recall are slightly worse than that in Table 2. The length of the inferred region is slightly longer. Most importantly, all the elements in the row "linked region recovery" are 1 for our program. This indicates that our program can always find the linked region that contains the mutation site. We can also see that our new program performs much better than the program in [6] in those cases. Comparing case 1 and case 2, we can see that case 2 is harder to handle. In case 2, the result for 2 children is very poor and this case is not solvable by our program.

**Table 3:** The experimental results for nuclear families when the genotype data of only one parent is available and this parent is diseased.

	2 children	3 children	4 children	5 children	6 children
No. of Breakpoints	3.68 (3.75)	7.47 (14.59)	10.32 (25.47)	12.90 (34.83)	15.48 (50.38)

precision	0.33 (0.39)	0.78 (0.26)	0.89 (0.23)	0.94 (0.24)	0.96 (0.21)

recall	0.23 (0.27)	0.72 (0.48)	0.86 (0.55)	0.91 (0.63)	0.92 (0.66)

length of real linked region (cM)	83.14	57.98	45.62	35.15	31.01

length of found linked region (cM)	111.1 (91.45)	65.08 (66.32)	48.85 (49.38)	37.40 (38.02)	32.57 (32.80)

overlap/real	1.00 (1.00)	1.00 (1.00)	1.00 (1.00)	1.00 (1.00)	1.00 (1.00)

overlap/found	0.86 (0.94)	0.93 (0.94)	0.94 (0.93)	0.93 (0.92)	0.93 (0.92)

linked region recovery	1.00 (255/285)	1.00 (229/285)	1.00 (267/285)	1.00 (276/285)	1.00 (281/285)

The meaning of each line is the same as in Table 2.

**Table 4:** The experimental results for nuclear families when the genotype data of only one parent is available and this parent is normal.

	3 children	4 children	5 children	6 children
No. of Breakpoints	7.39 (13.47)	10.29 (24.47)	12.94 (35.69)	15.23 (55.84)

precision	0.75 (0.31)	0.89 (0.25)	0.94 (0.24)	0.96 (0.19)

recall	0.68 (0.53)	0.86 (0.59)	0.90 (0.65)	0.92 (0.66)

length of real linked region (cM)	55.56	43.61	36.61	28.45

length of found linked region (cM)	67.66 (59.25)	43.90 (44.14)	36.87 (35.83)	29.23 (28.12)

overlap/real	0.99 (0.99)	0.99 (0.98)	0.99 (0.98)	0.99 (0.97)

overlap/found	0.85 (0.94)	0.99 (0.98)	0.99 (0.99)	0.98 (0.98)

linked region recovery	1.00 (275/285)	1.00 (274/285)	1.00 (274/285)	1.00 (266/285)

The meaning of each line is the same as in Table 2.

### Complicated pedigrees

**Figure 2:**
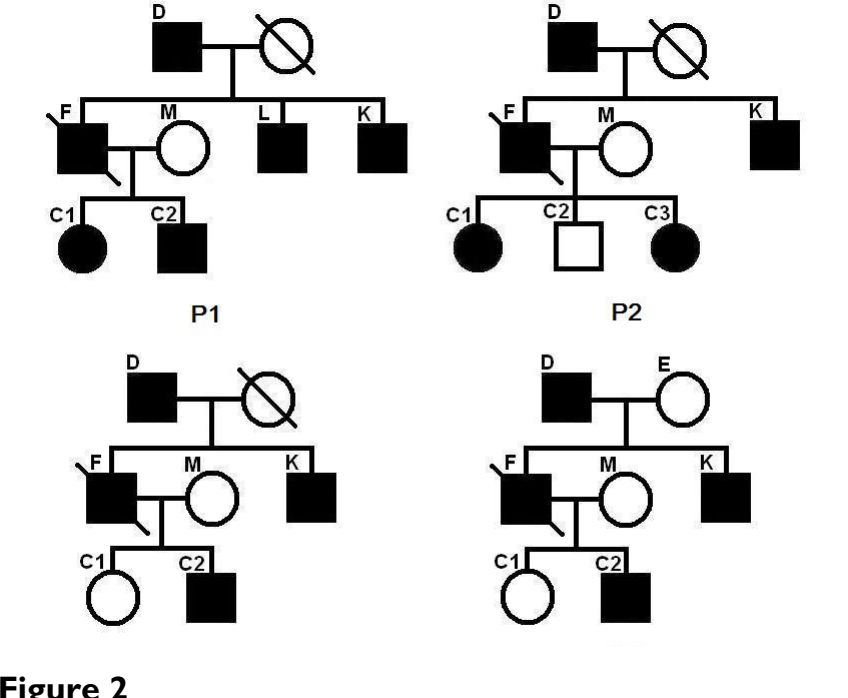
**Pedigrees P1 to P4**. Pedigrees P1 to P4. A slash on an individual indicates that the genotype data for this individual is missing.

We did experiments on some complicated pedigrees. First we study some pedigrees, where the genotype data for the super children in the second generation (shared by two nuclear families) is missing. See P1–P4 in Figure 2. A slash on an individual indicates that the genotype data for this individual is missing. This setting applies to all the figures in the paper.

**Figure 3:**
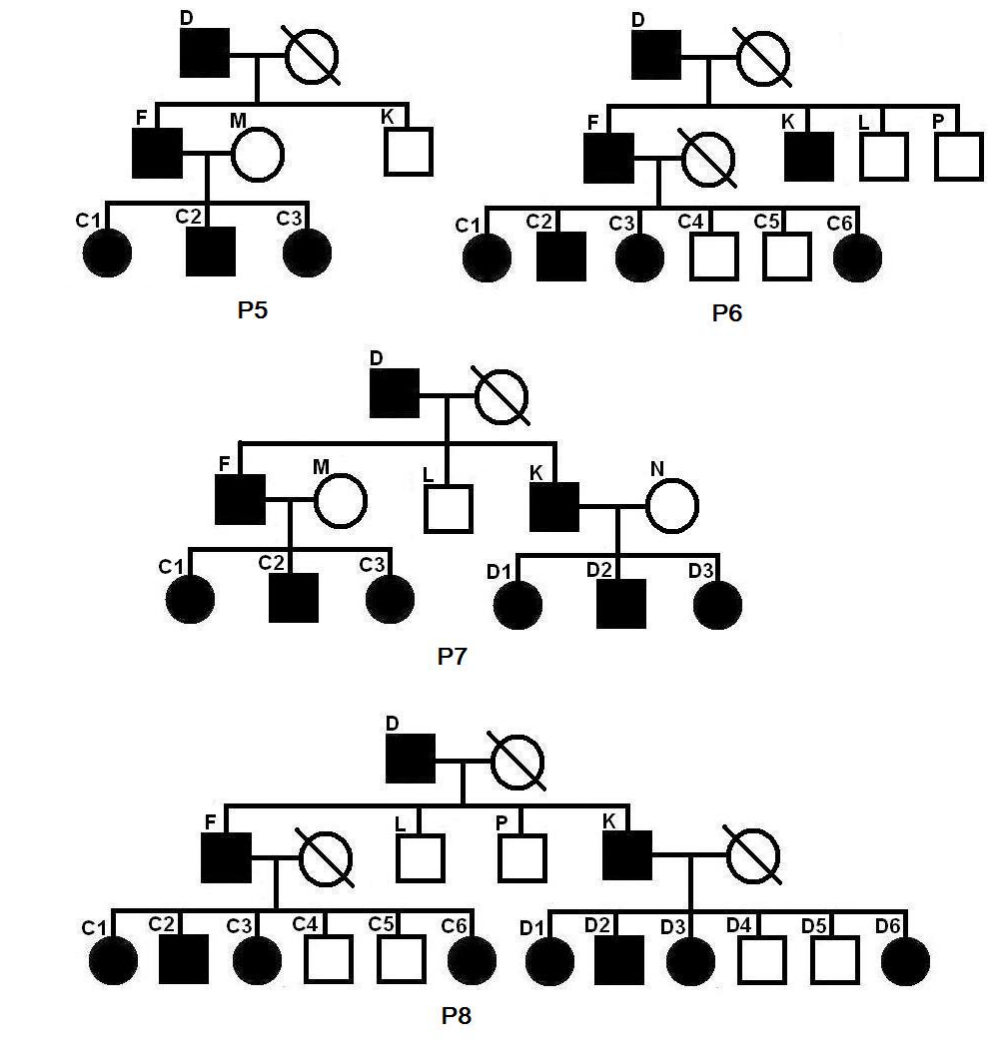
**Pedigrees P5 to P8**.

**Figure 4:**
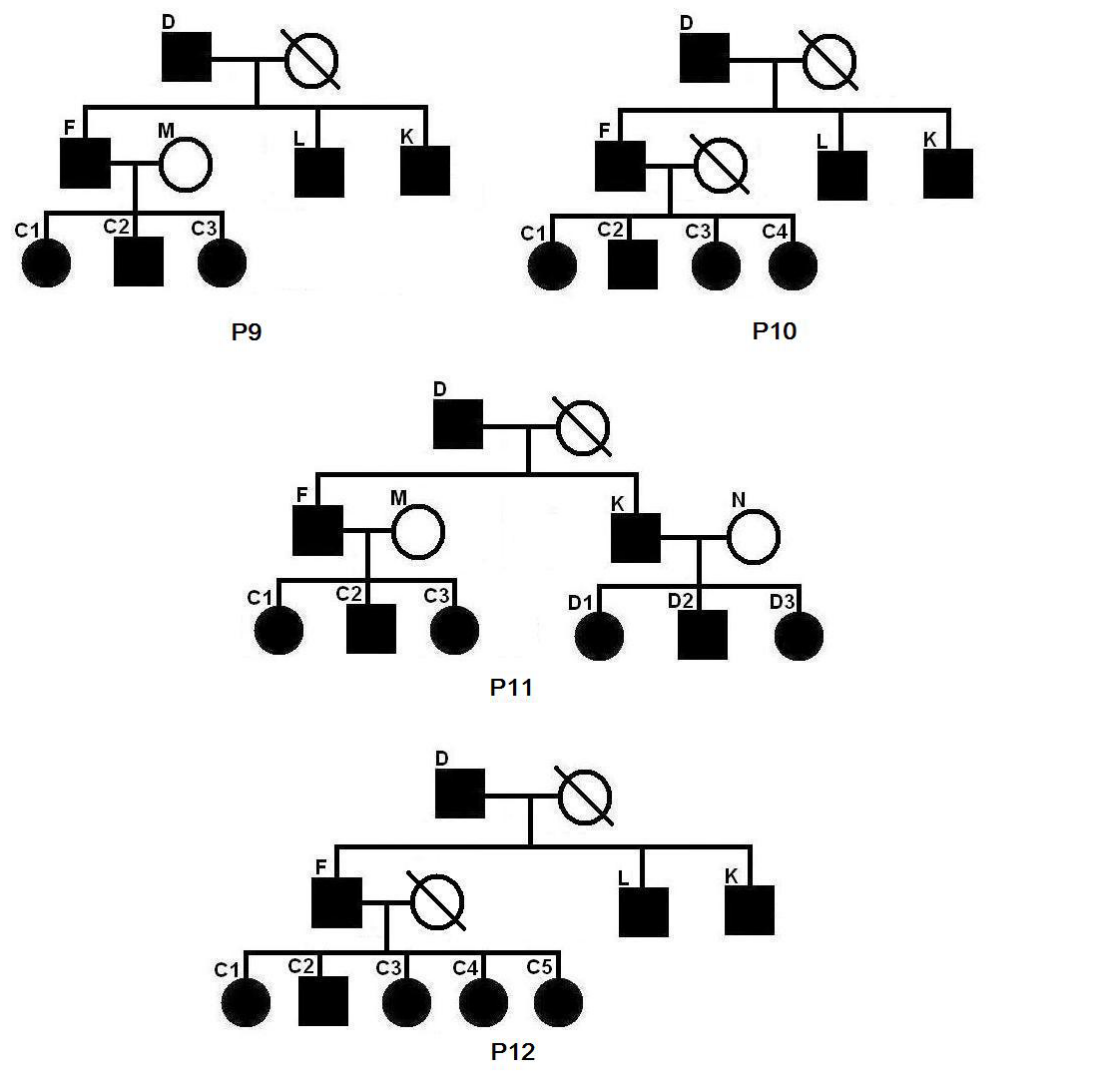
**Pedigrees P9 to P12**.

**Figure 5:**
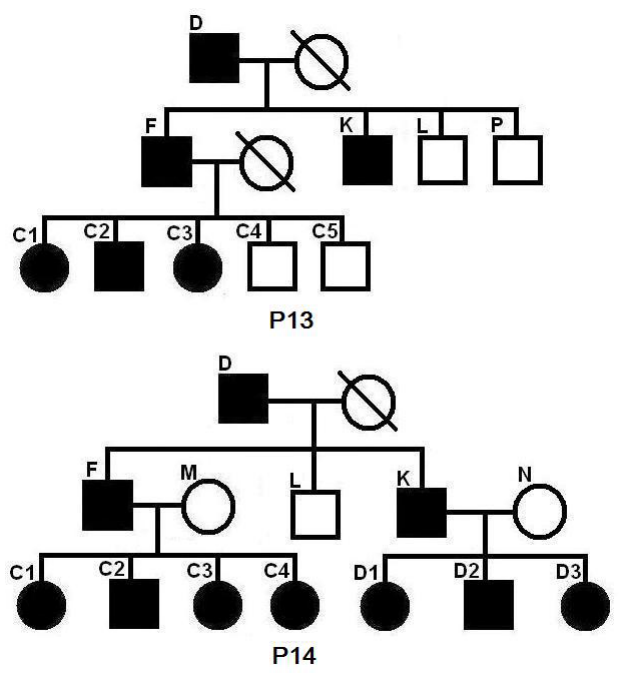
**Pedigrees P13 to P14**.

**Figure 6:**
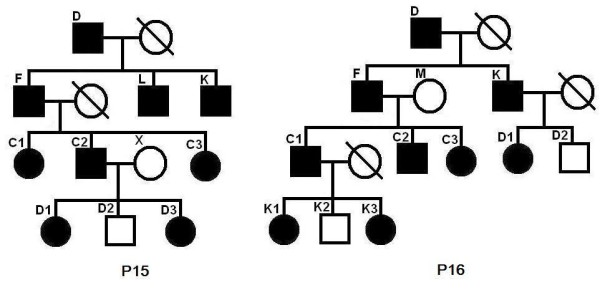
**Pedigrees P15 to P16**.

We have done 285 experiments on each of P1–P4. From Table 5, we can see that the inferred region of our program can almost always cover the entire real linked region. The length of the inferred linked region is a bit (about 15%) longer than that of the real linked region. From the row "linked region recovery", we can see that our program can always precisely find the linked region that contains the mutation site. Note that the program in [6] cannot handle P1–P4 at all. This is a significant improvement in our new program. Let us consider pedigrees P5–P8 in Figure 3, where individuals with missing genotype data are not super children (not shared by two nuclear families). We have done 285 experiments for each of the pedigrees. The results are listed in Table 6. From the row "linked region recovery", we can see that, if the nuclear family of the first generation has a single parent, and the nuclear family of the second generation has at least three (with both parents) or four (with single parent) children, we can always find the linked region that contains the mutation site. The program in [6] missed the mutation site at a rate of about 13.07%. Now we consider pedigrees P9–P16 in Figures 4, 5 and 6. The experimental results are shown in Table 7 and Table 8, respectively. We can see that for common pedigrees of different structures, our program can always find the linked region containing the mutation site and our program reports the linked region more precisely than the program in [6]. Note that the program in [6] sometimes missed the mutation sites. From all the listed experimental results, we can see that our program has much higher precision and recall than the program in [6], which indicates that our new program can infer the haplotypes more precisely.

**Table 5:** The experimental results for pedigrees P1 to P4.

	P1	P2	P3	P4
No. of Breakpoints	20.49	20.20	14.20	20.38

precision	0.58	0.77	0.49	0.57

recall	0.63	0.72	0.43	0.53

length of real linked region (cM)	34.69	36.42	43.89	41.28

length of found linked region (cM)	45.84	47.28	62.88	43.25

overlap/real	0.99	1	1	1

overlap/found	0.83	0.80	0.75	0.95

linked region recovery	1	1	1	1

The meaning of each line is the same as in Table 2.

**Table 6:** The experimental results for pedigrees P5 to P8.

	P5	P6	P7	P8
No. of Breakpoints	20.81 (31.60)	26.37 (47.27)	38.83 (108.4)	44.65 (145.4)

precision	0.86 (0.45)	0.96 (0.41)	0.97 (0.26)	0.90 (0.23)

recall	0.82 (0.66)	0.94 (0.71)	0.93 (0.71)	0.92 (0.76)

length of real linked region (cM)	38.58	19.77	21.92	12.37

length of found linked region (cM)	41.00 (43.28)	22.98 (23.51)	23.37 (24.57)	16.04 (16.20)

overlap/real	1.00 (1.00)	1.00 (1.00)	1.00 (1.00)	1.00 (1.00)

overlap/found	0.93 (0.93)	0.86 (0.84)	0.91 (0.91)	0.79 (0.78)

linked region recovery	1.00 (232/285)	1.00 (266/285)	1.00 (228/285)	1.00 (265/285)

The meaning of each line is the same as in Table 2.

**Table 7:** The experimental results for pedigrees P9 to P12.

	P9	P10	P11	P12
No. of Breakpoints	23.43 (41.07)	18.46 (26.38)	37.44 (92.49)	20.68 (31.52)

precision	0.95 (0.40)	0.92 (0.49)	0.91 (0.29)	0.94 (0.47)

recall	0.91 (0.67)	0.89 (0.68)	0.89 (0.70)	0.91 (0.71)

length of real linked region (cM)	29.80	26.06	22.26	22.05

length of found linked region (cM)	32.01 (36.72)	28.26 (31.93)	24.07 (24.53)	29.78 (30.57)

overlap/real	1.00 (1.00)	1.00 (1.00)	1.00 (1.00)	1.00 (1.00)

overlap/found	0.90 (0.81)	0.91 (0.83)	0.90 (0.89)	0.80 (0.78)

linked region recovery	1.00 (1.00)	1.00 (1.00)	1.00 (1.00)	1.00 (90/95)

The meaning of each line is the same as in Table 2.

**Table 8:** The experimental results for pedigrees P13 to P16.

	P13	P14	P15	P16
No. of Breakpoints	23.56 (46.66)	44.20 (124.0)	31.37 (48.25)	34.60 (69.26)

precision	0.97 (0.37)	0.98 (0.27)	0.96 (0.47)	0.88 (0.38)

recall	0.94 (0.71)	0.95 (0.75)	0.93 (0.71)	0.87 (0.75)

length of real linked region (cM)	21.36	18.22	21.41	18.40

length of found linked region (cM)	25.61 (25.91)	19.68 (20.42)	25.12 (27.60)	21.81 (22.39)

overlap/real	1.00 (1.00)	1.00 (1.00)	1.00 (1.00)	0.99 (1.00)

overlap/found	0.86 (0.84)	0.90 (0.89)	0.88 (0.81)	0.86 (0.85)

linked region recovery	1.00 (84/95)	1.00 (75/95)	1.00 (1.00)	1.00 (1.00)

The meaning of each line is the same as in Table 2.

### A Case Study

**Figure 7:**
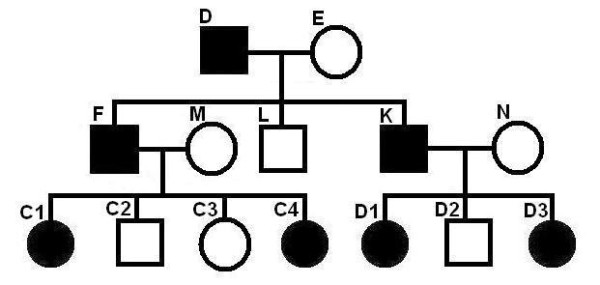
**A pedigree for case study**.

**Figure 8:**
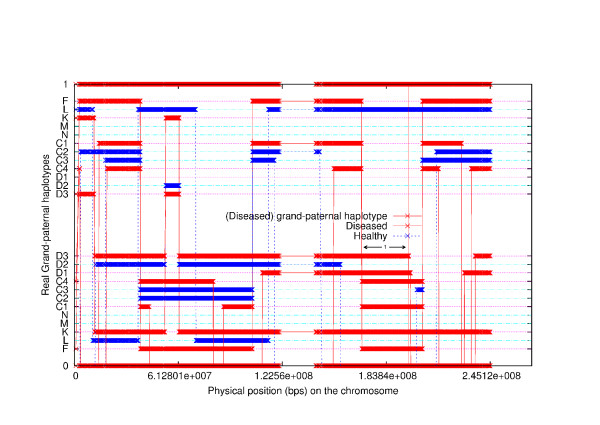
**The simulated grand-paternal haplotype allele sharing status among all members, excluding grandmother**. Each individual has two haplotypes, paternal haplotype and maternal haplotype. Note that individual D is the founder of the disease in the family. The two haplotypes of individual D are shown as 1-th haplotype and 0-th haplotype at the top and at the bottom, respectively. Any other individual may inherit alleles from both 0-th haplotype and 1-th haplotype of individual D. Thus, any other individual in the family appears twice (upper part and lower part) in the figure. The upper part shows the segments inherited from the 1-th haplotype of D and the lower part shows the segments inherited from the 0-th haplotype of D. The segments from diseased individuals are red and the segments from normal individuals are blue. The simulated (true) linked region is indicated by the horizontal black double-direction arrow in the middle.

**Figure 9:**
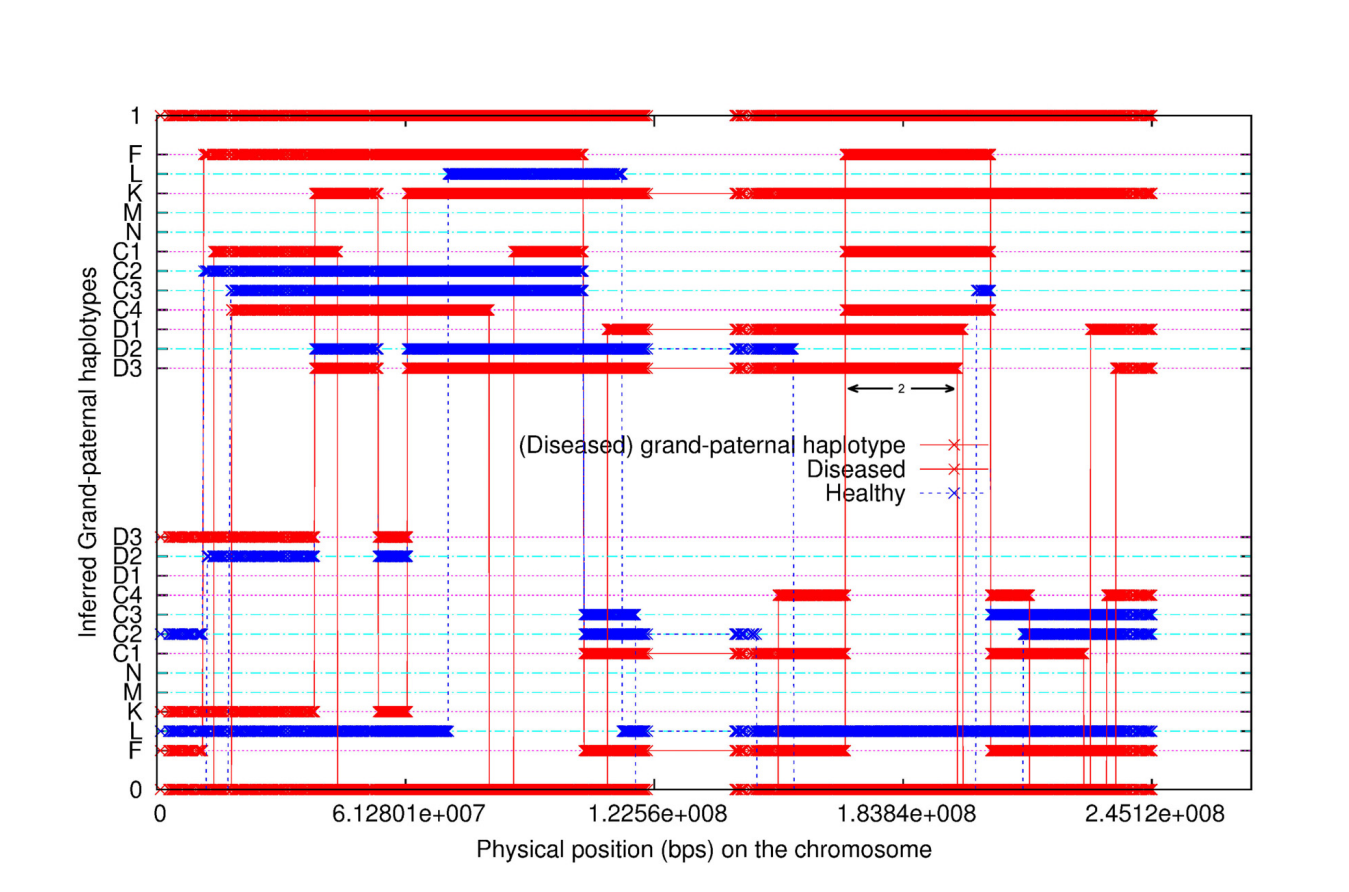
**The inferred grand-paternal haplotype allele sharing status among all members, excluding grandmother**.

We studied a pedigree to see if the program can correctly identify the linked region when some of the family members are purposely excluded. The pedigree is shown in Figure 7. The simulated allele sharing status and the linked region are shown in Figure 8. Each individual has two haplotypes, paternal haplotype and maternal haplotype. Note that individual D is the founder of the disease in the family. The two haplotypes of individual D are shown as 1-th haplotype and 0-th haplotype at the top and at the bottom, respectively. Any other individual may inherit alleles from both 0-th haplotype and 1-th haplotype of individual D. Thus, any other individual in the family appears twice (upper part and lower part) in the figure. The upper part shows the segments inherited from the 1-th haplotype of D and the lower part shows the segments inherited from the 0-th haplotype of D. The segments from diseased individuals are red and the segments from normal individuals are blue. At any position in the chromosome, each second generation individual gets an allele from D that is either from the 0-th haplotype or 1-th haplotype. For the third generation individual, say, C1, the allele at a position may or may not be from D. The simulated (true) linked region is from 189.61 cM to 211.55 cM (physical position from 169183745 bps to 197201161 bps) indicated by the horizontal black double-direction arrow in the middle. The inferred configuration is shown in Figure 9. We can see that the inferred configuration is roughly the same as the simulated configuration except that it is upside down. The inferred linked region is from 189.61 cM(169174855 bps) to 211.63 cM(197295627 bps). The inferred linked region remains the same if we exclude individual E or F. The inferred linked region also remains unchanged if we simultaneously remove E, M and N. When we simultaneously remove E, K, N, D1, D2 and D3, the inferred linked region is from 189.61 cM to 225.48 cM, which is only slightly enlarged. When we simultaneously remove E, F, M, C1, C2, C3 and C4, the inferred linked region is again enlarged and the region is from 172.07 cM to 211.63 cM. When F, K, N, D1, D2 and D3 are simultaneously removed from the pedigree, the inferred linked region is from 189.61 cM and 221.90 cM.

### Genotype data error correction

To test the effect of genotype data errors on our program, we did experiments on the pedigree in Figure 7. We generated genotype errors by randomly changing the genotype value (which is one of AA, BB and AB) into a different value (which is one of the other two values) at a position with probability 0.1% and 0.5%, respectively. We simulated 475 data sets. The length of real linked region ranges from 0.76 cM to 65.17 cM. The experimental results are shown in Table 9. For each cell, there are two values. The first one is by our program and the one in brackets is by the program in [6]. We can see that our program performs better and can always recover the real linked regions.

**Table 9:** The experimental results for genotype data error correction using Affymetrix 50 K GeneChips data.

	precision	recall	linked region recovery	overlap/real	overlap/found
No error	97.74%	95.05%	100%	99.98%	92.46%
	(69.62%)	(86.56%)	(100%)	(100%)	(91.78%)

0.1% error	84.40%	94.94%	100%	99.93%	92.00%
	(67.19%)	(86.19%)	(100%)	(100%)	(91.13%)

0.5% error	79.00%	93.76%	100%	99.84%	92.57%
	(61.81%)	(86.10%)	(100%)	(100%)	(91.94%)

### Comparison with Merlin

We compared our program with Merlin [16]. We did the experiments on a PC with a CPU of 3.0 GHz and 1.00 GB memory. The results are shown in Table 10 and Table 11 for Affymetrix 50 K GeneChips and Affymetrix 250 K GeneChips, respectively. We have also considered different kinds of pedigrees.

**Table 10:** Comparison with Merlin using Affymetrix 50 K GeneChips.

	Run time (s)	overlap/real	overlap/found
0+3	16.717 (0.898)	0.941 (0.805)	0.605 (0.862)

0+4	23.325 (1.126)	0.928 (0.824)	0.594 (0.930)

0+5	30.634 (1.746)	0.941 (0.901)	0.587 (0.936)

0+6	39.028 (3.901)	0.987 (0.929)	0.576 (0.969)

2+3(4 bits)	3.276 (1.340)	1.000 (0.928)	0.960 (1)

2+4(6 bits)	4.596 (1.735)	0.9997 (0.937)	0.967 (0.990)

P5(7 bits)	7.714 (2.325)	0.999 (0.933)	0.919 (0.979)

P9(9 bits)	7.365 (3.255)	0.999 (0.879)	0.926 (0.958)

P10(11 bits)	19.15 (8.829)	0.999 (0.797)	0.912 (0.968)

P11(12 bits)	11.37 (14.01)	1.000 (0.938)	0.924 (0.979)

P12(13 bits)	25.62 (31.57)	1.000 (0.922)	0.842 (0.958)

P7(14 bits)	13.58 (58.14)	1.000 (0.949)	0.933 (0.990)

P15(14 bits)	46.96 (43.15)	1.000 (0.906)	0.894 (0.979)

P13(15 bits)	23.72 (1282.05)	1.000 (0.970)	0.875 (1)

P16(15 bits)	37.93 (384.78)	0.988 (0.950)	0.877 (0.990)

P14(16 bits)	15.042 (NA)	1.000 (NA)	0.927 (NA)

P6(17 bits)	40.754 (NA)	1.000 (NA)	0.869 (NA)

Each cell contains two values. The first one is by our program and the one in brackets is by Merlin. Pedigree 0+i for i = 3, 4, 5 and 6 stands for a nuclear family with i children and the genotype data for parents is not available. Pedigree 2+i for i = 3 and 4 stands for a nuclear family with i children and the genotype data for both parents is available. "NA" means that the program failed to execute on this pedigree.

**Table 11:** Comparison with Merlin using Affymetrix 250 K GeneChips.

	Run time (s)	overlap/real	overlap/found
0+3	67.680 (2.979)	0.954 (0.975)	0.590 (0.866)

0+4	97.082 (4.067)	0.965 (0.988)	0.548 (0.955)

0+5	124.43 (6.558)	0.983 (0.994)	0.607 (0.960)

0+6	131.71 (12.50)	0.994 (0.992)	0.675 (0.986)

2+3(4 bits)	9.454 (7.122)	1 (0.936)	0.995 (1)

2+4(6 bits)	11.625 (8.133)	1 (0.989)	0.988 (1)

P5(7 bits)	16.662 (8.262)	1 (0.892)	0.983 (0.938)

P9(9 bits)	24.18 (16.65)	1.000 (0.934)	0.973 (1)

P10(11 bits)	38.54 (31.39)	1.000 (0.797)	0.970 (0.938)

P11(12 bits)	29.94 (415.18)	1.000 (0.991)	0.972 (1)

P12(13 bits)	49.28 (1237.7)	1.000 (0.968)	0.871 (1)

P7(14 bits)	31.611 (NA)	1(NA)	0.960(NA)

P15(14 bits)	103.269 (NA)	1(NA)	0.927(NA)

P13(15 bits)	54.114 (NA)	1.000(NA)	0.881(NA)

P16(15 bits)	141.266 (NA)	0.991(NA)	0.922(NA)

P14(16 bits)	52.911 (NA)	1(NA)	0.979(NA)

P6(17 bits)	63.984 (NA)	1.000(NA)	0.862(NA)

Each cell contains two values. The first one is by our program and the one in brackets is by Merlin. Pedigree 0+i for i = 3, 4, 5 and 6 stands for a nuclear family with i children and the genotype data for both parents is not available. Pedigree 2+i for i = 3 and 4 stands for a nuclear family with i children and the genotype data for both parents is available. "NA" means that the program failed to execute on this pedigree.

#### Running time

For small sized families, both programs can generate results in a few seconds. When the sizes of the families and the number of the markers increase, the running time of our program increases linearly. For large families, Merlin requires really long running time. Most importantly, Merlin needs large memory space for big sized families and cannot successfully complete the computation for some pedigrees (see P14 and P6 in Table 10 and Table 11).

#### Output Quality

We again use "overlap/real" and "overlap/found" to indicate the quality of the computational results. Our program always clearly gives a computed candidate region for each input. Merlin calculates a LOD score for each marker. We evaluated the segment with the highest LOD score as the output linked region for Merlin. From Table 10 and Table 11, we can see that the results of our program are less than optimal when parental data is not available at all. When the family size becomes bigger, our program outperforms Merlin. Our program can quickly produce accurate linked regions when the family size is big while Merlin failed to execute on big sized families.

## Conclusion

We have developed a software package that infers the haplotype allele sharing status for the members of a pedigree based on the minimum recombinants model. The running time of the program is linear in terms of the input size *O*(*mn*), where *m *is the total number of individuals in the whole family and *n *is the number of SNP sites in the chromosome. The new package can handle a wide range of pedigree structures. It works very well for cases where the genotype data of one parent is missing for the entire chromosome.

## Availability and requirements

**Project Name**: CityU 121608

**Project Homepage**: 

**Operating system(s)**: Platform independent

**Programming language**: Java

**Other requirements**: Java 1.6.0 or higher

**Licence**: None

**Any restrictions to use by non-academics**: None

## Authors' contributions

LW carried out the algorithm design, provided some of initial pseudo codes, checked part of computer codes, participated in the design of experiments, and drafted the manuscript. ZW produced the computer program, carried out the experiments, drafted experiment results, and participated in algorithm design. WY initiated the study, participated in the design of experiments, polished the manuscript, and was in charge of communication with the editor. All the authors read and approved the final manuscript.
